# White Matter Microstructure is Associated with Auditory and Tactile Processing in Children with and without Sensory Processing Disorder

**DOI:** 10.3389/fnana.2015.00169

**Published:** 2016-01-26

**Authors:** Yi-Shin Chang, Mathilde Gratiot, Julia P. Owen, Anne Brandes-Aitken, Shivani S. Desai, Susanna S. Hill, Anne B. Arnett, Julia Harris, Elysa J. Marco, Pratik Mukherjee

**Affiliations:** ^1^Department of Radiology and Biomedical Imaging, University of California, San FranciscoSan Francisco, CA, USA; ^2^Department of Cognitive Neuroscience, Université Pierre et Marie CurieParis, France; ^3^Department of Neurology, School of Medicine, University of California, San FranciscoSan Francisco, CA, USA; ^4^Department of Psychiatry, University of California, San FranciscoSan Francisco, CA, USA; ^5^Department of Pediatrics, University of California, San FranciscoSan Francisco, CA, USA

**Keywords:** diffusion tensor imaging, sensory processing disorders, auditory processing, tactile processing, white matter

## Abstract

Sensory processing disorders (SPDs) affect up to 16% of school-aged children, and contribute to cognitive and behavioral deficits impacting affected individuals and their families. While sensory processing differences are now widely recognized in children with autism, children with sensory-based dysfunction who do not meet autism criteria based on social communication deficits remain virtually unstudied. In a previous pilot diffusion tensor imaging (DTI) study, we demonstrated that boys with SPD have altered white matter microstructure primarily affecting the posterior cerebral tracts, which subserve sensory processing and integration. This disrupted microstructural integrity, measured as reduced white matter fractional anisotropy (FA), correlated with parent report measures of atypical sensory behavior. In this present study, we investigate white matter microstructure as it relates to tactile and auditory function in depth with a larger, mixed-gender cohort of children 8–12 years of age. We continue to find robust alterations of posterior white matter microstructure in children with SPD relative to typically developing children (TDC), along with more spatially distributed alterations. We find strong correlations of FA with both parent report and direct measures of tactile and auditory processing across children, with the direct assessment measures of tactile and auditory processing showing a stronger and more continuous mapping to the underlying white matter integrity than the corresponding parent report measures. Based on these findings of microstructure as a neural correlate of sensory processing ability, diffusion MRI merits further investigation as a tool to find biomarkers for diagnosis, prognosis and treatment response in children with SPD. To our knowledge, this work is the first to demonstrate associations of directly measured tactile and non-linguistic auditory function with white matter microstructural integrity – not just in children with SPD, but also in TDC.

## Introduction

Hypo-and/or hyper responsiveness to sensory stimulation is estimated to occur in 5–16% of children within the general population, and 40–80% of children with neurodevelopmental disorders (Ahn et al., 2004). Such sensory dysfunction can hinder a child’s ability to accomplish practical, daily activities and age-appropriate learning tasks, thus resulting in long-term impairment of intellectual and social abilities. Sensory processing disorder (SPD) is reported to be highly co-incident with attention deficit hyperactivity disorder (ADHD) and autism spectrum disorders (ASDs). However, it is also clear that children can have sensory processing dysfunction without the degree of attention, language, or social challenges that would meet criteria for ADHD or ASD. This has been referred to in the literature as isolated SPD. The Diagnostic Classification of Mental Health and Developmental Disorders in Infancy and Early Childhood includes a diagnostic label for Regulation Disorders of Sensory Processing (Zero to Three, 2005), but the Diagnostic and Statistical Manual 5 (DSM-V) does not include SPDs as a standalone category. They do now, however, include hyper- or hyporeactivity to sensory input or unusual interest in sensory aspects of the environment in their revised ASD criteria. These sensory processing differences are being increasingly investigated in the field of autism research and recognized as a core and critical clinical feature—however, children with SPD who do not also have social communication deficits that meet ASD criteria can provide insight into the neural underpinnings of sensory processing in particular.

The present literature on SPD primarily utilizes parent/caregiver report measures that describe sensory-related behaviors and physiological measures that provide information about arousal and sensory reactivity. Recently, our group has published two studies using diffusion tensor imaging (DTI) to better define the neural correlates of these sensory processing deficits. Our first study took a whole-brain, data-driven approach to demonstrate decreased fractional anisotropy (FA) and increased mean diffusivity (MD) and radial diffusivity (RD), reflecting reduced microstructural integrity, in the posterior white matter tracts of 16 boys with SPD compared to 24 neurotypically developing boys (Owen et al., 2013). In addition, we found that FA in affected brain regions correlated with atypical auditory, multisensory, and attention-related behaviors as reported by parents on the Sensory Profile. In a subsequent report, we established that children with SPD and those with ASD both demonstrate decreased FA in parietal-occipital tracts whereas only children with ASD show differences in temporal tracts subserving social-emotional processing (Chang et al., 2014). We recently investigated whether direct assessment measures of tactile and auditory processing might also inform our understanding and evaluation of children with atypical sensory related behaviors both with and without an ASD diagnosis. We found that children with both ASD and SPD show impairment in tactile processing (right hand graphesthesia) whereas only the ASD group showed significant impairment in a measure of cortical auditory processing (Demopoulos et al., 2015). However, correlations including all children (controls, SPD, and ASD) showed a significant association between a direct measure of auditory processing impairment and a parent report measure of real-world communication ability.

As our previous imaging findings were limited to a small cohort of affected boys, we seek to investigate these results in a larger mixed-gender cohort sample. We hypothesize that boys and girls with SPD will show impaired white matter microstructural integrity, with a posterior predominance, relative to typically developing children (TDC). We further hypothesize that this microstructural integrity will correlate with parent report as well as with direct measurements of sensory processing, but that the direct measurements will show stronger correlation with the underlying microstructure.

## Materials and Methods

### Demographic, Sensory, Cognitive and Behavioral Data

Children ages 8–12 years were enrolled under an institutional review board approved protocol. SPD subjects were recruited from the UCSF Sensory Neurodevelopment and Autism Program (SNAP) and from local online parent board listings. TDC were recruited from online parent group listings as well as referrals from affiliated sensory neurodevelopment and autism research groups. Informed consent was obtained from the parents or legal guardians, with the assent of all participants. Exclusion criteria were brain malformation or injury, movement disorder, bipolar disorder, psychotic disorder, hearing impairment, full-scale IQ (FSIQ) score <70 on the Wechsler Intelligence Scale for Children-Fourth Edition (WISC-IV; Wechsler, 2003), or meeting criteria for ASD. Subjects were also excluded for any anomalies or artifacts on MR imaging or DTI using criteria explained below in Section “DTI Analysis.” A total of 40 right-handed children with SPD (32 male, 8 female) and 41 right-handed TDC (28 male, 13 female) met all inclusion and exclusion criteria for the study.

Autism spectrum disorders was intially screened for using the Social Communication Questionnaire (SCQ; Rutter et al., 2003), a parent report screening measure for autism symptoms, with a score of 15 or above being suggestive of ASD. There were four boys in the SPD cohort who exceeded the SCQ screening cut off score of 15, which prompted a standardized structured play session following the Autism Diagnostic Observation Schedule (ADOS; Lord et al., 2000) and clinical review. All four boys scored less than 3 for the social and communication total score with a score greater than 7 meeting concern for ASD and a score greater than 10 meeting concern for a full autism disorder diagnosis based on DSM IV-TR criteria. Clinical interview and review by Dr. Marco, a pediatric cognitive and behavioral child neurologist was also non-consistent with a clinical ASD diagnosis. None of the TDC cohort and none of the SPD girls met screening criteria for ASD based on the SCQ.

All TDC and SPD subjects were assessed with the Sensory Profile (Dunn and Westman, 1997), a parent report questionnaire which measures atypical sensory related behaviors. A definite difference in each sensory domain is defined as a score that is greater than two standard deviations from the mean: ≤25 out of 40 for auditory processing, ≤64 out of 90 for tactile processing, ≤26 out of 45 for visual processing, and ≤23 out of 35 for multisensory integration. There was one child without an auditory processing score, one without tactile, one without multisensory, two without visual scores, and five without sensory profile total scores given incomplete data from the parent survey. A child was included in the SPD cohort if they carried an outside diagnosis of SPD from a community occupational therapist and they had a score in the definite difference range on at least one of the Sensory Profile subscores listed above. Six of the TDC group scored in the probable difference range for one of the subscales, while the remainder scored in the normative range on all subscales. Finally, two SPD kids and one control were born early (gestational ages 36, 35, 33 weeks, respectively).

The Acoustic Index of the Differential Screening Test for Processing (DSTP) was used to assess auditory processing (Richard and Ferre, 2006) for all subjects. This test was administered by the lead study coordinator in accordance with manualized instructions and with training and supervision by a cognitive and behavioral neurologist. The acoustic index is calculated from totaling correct items in: (1) dichotic listening, an index of interhemispheric auditory processing, in which the participant hears and repeats different numbers simultaneously presented through headphones to each ear; (2) temporal patterning, in which the participant reports the order of high and low tones presented in a sequence; and (3) auditory discrimination, in which a participant repeats nonsense syllables presented in background noise. Thirty four out of the 41 controls and 35 out of the 40 SPD subjects received the DSTP. The graphesthesia subtest of The Sensory Integration Praxis Tests (Ayres, 1989) was used to assess tactile proprioception by asking participants to recreate seven designs (neither numbers nor letters) drawn on the dorsum of each hand with closed eyes. Each drawing is then scored for accuracy. This test was administered by the lead study coordinator in accordance with manualized instructions and with training and supervision by a cognitive and behavioral neurologist. 33 out of the 41 controls and 34 out of the 40 SPD subjects received the graphesthesia assessment.

Summary demographic information is included in **Table 1**. No significant differences were found in any of the demographic or sensory variables between boys and girls, for either TDC or SPD kids.

**Table 1:** Demographic information and sensory scores.

	#TDC/#SPD	TDC (mean ± standard deviation)	SPD (mean ± standard deviation)	*p*
Age (years)	41/40	10.1 ± 1.1	9.6 ± 1.2	0.066
FSIQ	41/40	116 ± 10	112 ± 13	0.077
SP – Auditory	41/39	33.8 ± 3.5	23.2 ± 5.00	**1.2E-16**
SP – Tactile	41/39	83.5 ± 5.8	62.4 ± 12.2	**1.5E-13**
DSTP	34/35	36.1 ± 3.53	32.2 ± 5.6	**0.0017**
Graphesthesia	33/34	21.5 ± 3.91	19.1 ± 4.8	**0.0025**

*Bolded p values indicate statistically significant group differences using an unpaired student t-test (p < 0.05).*

### Image Acquisition

MR imaging was performed on a 3T Tim Trio scanner (Siemens, Erlangen, Germany) using a 12 channel head coil. Structural MR imaging of the brain was performed with an axial 3D magnetization prepared rapid acquisition gradient-echo T1-weighted sequence (TE = 2.98 ms, TR = 2300 ms, TI = 900 ms, flip angle of 90°) with in-plane resolution of 1 × 1 mm on a 256 × 256 matrix and 160 1.0 mm contiguous partitions. Whole-brain diffusion imaging was performed with a multislice 2D single-shot twice-refocused spin echo echo-planar sequence with 64 diffusion-encoding directions, diffusion-weighting strength of *b* = 2000 s/mm^2^, iPAT reduction factor of 2, TE/TR = 109/8000 ms, NEX = 1, interleaved 2.2 mm-thick axial slices with no gap, and in-plane resolution of 2.2 mm × 2.2 mm on a 100 × 100 matrix. An additional image volume was acquired with no diffusion weighting (*b* = 0 s/mm^2^). The total diffusion acquisition time was 8.7 min. Structural MRI for all children was reviewed by Dr. Pratik Mukherjee, a pediatric neuroradiologist, who was blind to cohort assignment. No structural anomalies or other clinically significant findings were reported.

### DTI Analysis

#### Pre-processing

The diffusion-weighted images were corrected for motion and eddy currents using FMRIB’s Linear Image Registration Tool (FLIRT^1^) with 12-parameter linear image registration (Jenkinson et al., 2002). All diffusion-weighted volumes were registered to the reference *b* = 0 s/mm^2^ volume. To evaluate subject movement, we calculated a scalar parameter quantifying the transformation of each diffusion volume to the reference. Sixteen children (6 TDC, 10 SPD) were excluded for DTI artifacts and/or median relative displacement between volumes greater than 2 mm, where a volume represents a single diffusion directional measurement of the entire brain. This left a total of 81 children (40 SPD, 41 TDC) with DTI datasets meeting quality control criteria. A heteroscedastic two-sample Student’s *t*-test verified that there were no significant differences between these SPD and TDC groups in movement during the DTI scan (*p* > 0.05). The non-brain tissue was removed using the Brain Extraction Tool (BET^2^). FA, mean diffusivity (MD), and radial diffusivity (RD) were calculated using FSL’s DTIFIT at every voxel, yielding FA, MD, and RD maps for each subject.

#### Group Differences

Tract-Based Spatial Statistics (TBSS) in FSL (Smith et al., 2006) was used to skeletonize and register the diffusion maps for each subject in order to perform voxel-wise comparisons along the white matter skeleton. First, each subject’s FA map was non-linearly registered to each other subject’s FA map to identify the most representative FA map as a registration target. The registered maps were then averaged and skeletonized to the center of the white matter. Next, each subject’s FA data was projected onto this mean skeleton to obtain skeletonized FA maps per subject. MD and RD maps were then registered and projected onto the white matter skeleton. Finally, voxelwise statistics were performed on the skeletonized maps to assess for group differences with non-parametric permutation testing using the *randomize* function from FSL. Based on our prior work (Owen et al., 2013), we tested the contrasts of: TDC > SPD for FA, SPD > TDC for MD, and SPD > TDC for RD. The resulting group difference maps for each comparison were corrected for multiple comparisons over the 3D image volume with threshold-free cluster enhancement (TFCE) (Smith and Nichols, 2009) using a significance threshold of *p* < 0.05. It is important to note that, in TFCE, the cluster, and not the individual voxel, is the ultimate object of statistical inference and therefore every voxel in the cluster has exactly the same level of statistical significance in the final results. The Johns Hopkins University (JHU) ICBM-DTI-81 White-Matter Labeled Atlas (Wakana et al., 2004) was used to determine the anatomic locations of white matter regions.

#### Effects of Age and Gender

For each subject, mean FA values were obtained within the significant voxels of each of the four white matter regions implicated in the group difference analyses – the left and right posterior thalamic radiations (PTR), splenium of the corpus callosum (SCC), and retrolenticular limb of the right internal capsule (RLIC), as defined by the JHU white matter atlas. Then, general linear models (GLMs) of FA as a function of group (TDC or SPD), age, and gender were created for the each of these ROIs:

FAroi=β0+β1group*+β2age*+β3gender*

where group is 1 for TDC and 0 for SPD, and gender is 1 for females and 0 for males. The coefficient values (β_1_-β_3_) and their significance levels were assessed for each ROI.

#### Sensory Correlations with DTI

In order to limit the number of statistical comparisons, FA alone was examined for correlations with our sensory variables. FA was tested for correlations with the Sensory Profile auditory score and DSTP separately to assess the relationship between white matter microstructure and auditory processing. FA was tested for correlations with the Sensory Profile tactile score and Graphesthesia separately to assess the relationship between white matter microstructure and tactile processing. The correlation analyses were performed on a voxel-wise basis along the white matter skeleton. *Randomize* was used to assess for significant positive correlations of FA with each parent report or direct assessment, with regression of the motion parameters, and the resultant statistical maps were corrected for multiple comparisons using TFCE with *p* < 0.05.

As a *post hoc* analysis to determine contributions of group, age, and gender to the correlational results, for each sensory metric, FA was averaged across the voxels of significant clusters separately for each subject, and separately for several JHU white matter regions. Then, GLMs were constructed with the cognitive metric as the response variable, and FA, group (TDC vs. SPD), age, and gender as predictor variables. The significance of each predictor variable for each model was determined.

To further investigate the contributions of different types of auditory processing to the correlational results found with DSTP, an additional *post hoc* analysis of correlations between the three subscores of the DSTP acoustic subtest and FA was conducted using the same approach described above.

## Results

### Group Differences

**FIGURE 1:**
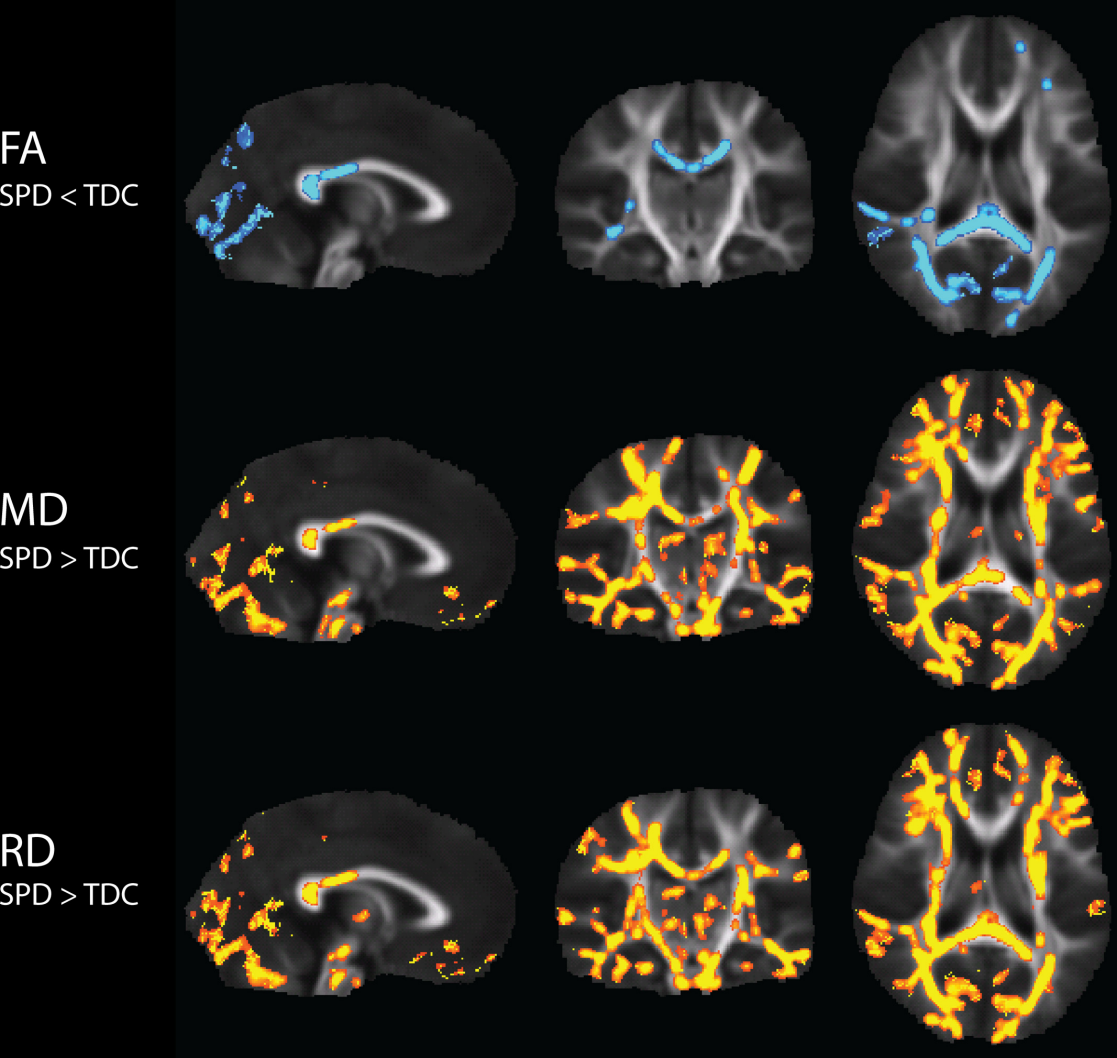
**Tract-Based Spatial Statistics (TBSS) results of group differences in FA, MD, and RD between TDC and SPD.** Blue regions indicate voxels of significant decreases in SPD relative to TDC, while yellow regions indicate voxels of significant increases in SPD relative to TDC.

Significantly lower FA, and higher MD and RD values, were found in the SPD cohort relative to TDC (**Figure 1**). Visual reference with the JHU white matter atlas reveals that the differences in FA are primarily localized to the bilateral PTRs, the SCC, and the right RLIC. MD and RD showed extensive elevations throughout the white matter. Consistent with the FA results, MD and RD are elevated only in the posterior corpus callosum. Also consistent with the FA results, the most significant elevations of both MD and RD occur in the bilateral PTR and right RLIC.

### Effects of Age and Gender

The coefficient estimates and *p*-values for the GLM of FA as a function of group, age, and gender, are displayed in **Table 2**. The effect of group remained strongly significant when accounting for age and gender. As expected, increasing age contributed to higher FA in the bilateral PTR and SCC. There were no significant gender effects.

**Table 2:** Coefficient estimates and *p*-values for the general linear model of FA in a few significant regions as a function of group, age, and gender.

	Group (TDC = 1, SPD = 0)		Age		Gender (*F* = 1, *M* = 0)	
	b1	*p*	b2	*p*	b3	*p*
PTR-L	0.028	**0.00034**	0.0066	**0.038**	0.012	0.14
PTR-R	0.028	**0.00053**	0.0080	**0.017**	0.016	0.063
RLIC-R	0.014	**0.011**	0.0033	0.16	0.010	0.12
SCC	0.019	**0.00033**	0.0049	**0.022**	0.0039	0.49

*PTR, posterior thalamic radiation; RLIC, retrolenticular limb of the internal capsule; SCC, splenium of the corpus callosum. Bolded p values indicate statistically significant coefficient estimates (p < 0.05).*

### Sensory Correlations with DTI

#### Tactile Correlations with DTI

**FIGURE 2:**
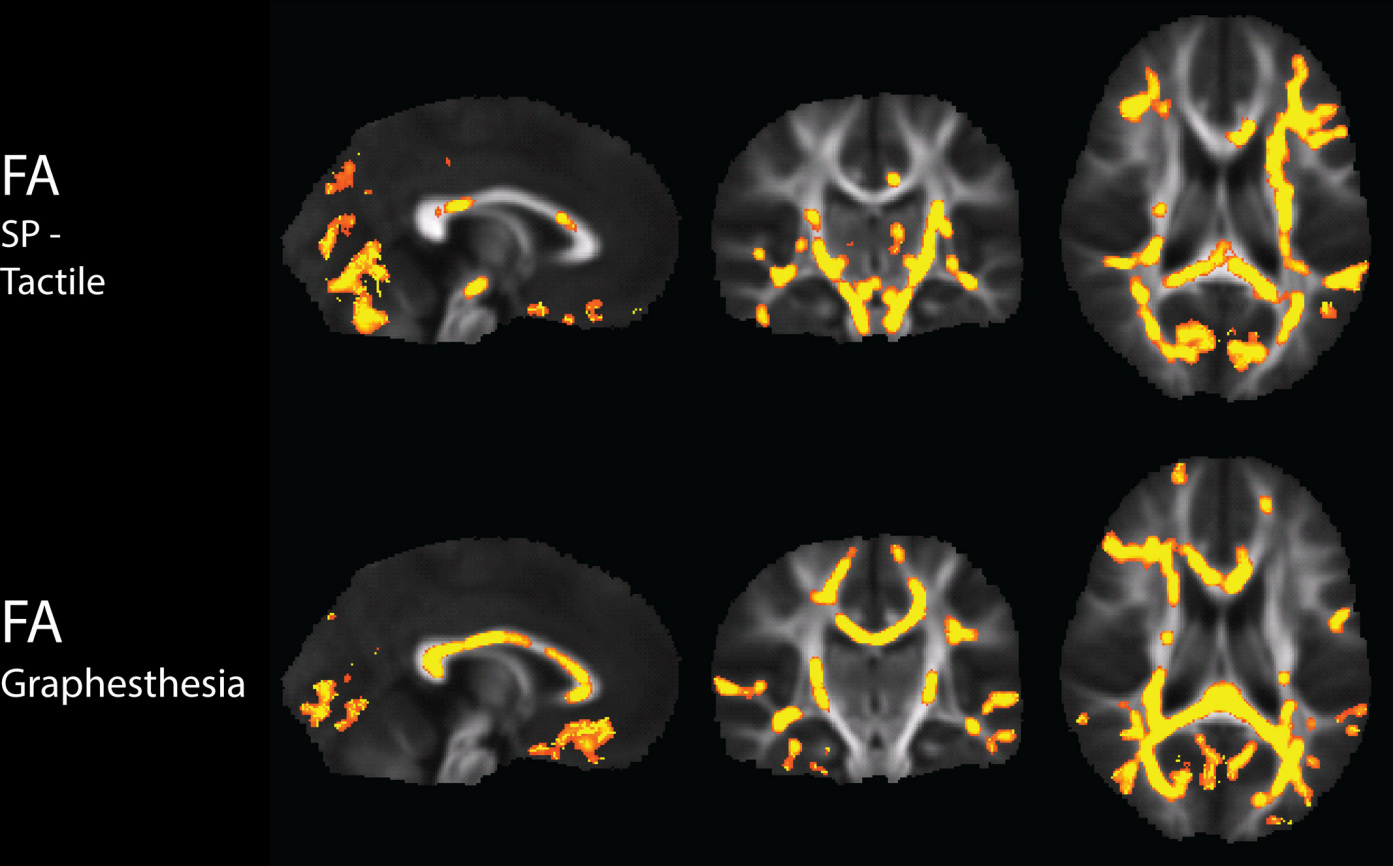
**Tract-Based Spatial Statistics results of correlations of the Sensory Profile tactile score and Graphesthesia with FA, including regression of motion**.

There were widespread, significant positive associations of FA across groups with the Sensory Profile tactile score and with graphesthesia, after regression of motion parameters (**Figure 2**). The number of significantly correlated voxels in several ROIs is included in **Table 3**.

**Table 3:** Number of significantly correlated voxels in several ROIs, along with results of the GLMs of Sensory Profile tactile score and Graphesthesia as functions of group, FA, age, and gender.

	SP Tactile			Graphesthesia		
	# sig vox	p_FA	p_TDCvSPD	# sig vox	p_FA	p_TDCvSPD
ACR-L	659	0.074	**4.1E-13**	361	0.11	**0.024**
ACR-R	372	0.14	**2.0E-12**	938	**0.039**	**0.020**
SCR-L	179	**0.0037**	**5.6E-15**	181	**0.034**	**0.012**
SCR-R	24	0.85	**9.0E-14**	170	**0.0072**	**0.0080**
PCR-L	221	**0.031**	**8.4E-14**	232	**0.023**	**0.012**
PCR-R	229	0.11	**1.8E-13**	398	0.056	**0.031**
ALIC-L	267	0.29	**9.0E-13**	–	–	–
ALIC-R	308	0.10	**2.3E-12**	234	**0.014**	**0.034**
PLIC-L	590	**0.014**	**5.9E-14**	49	0.20	**0.0077**
PLIC-R	506	**0.012**	**2.1E-13**	322	**0.010**	**0.016**
RLIC-L	393	**0.033**	**3.9E-13**	375	0.721	**0.011**
RLIC-R	333	0.091	**3.4E-13**	386	0.301	**0.021**
PTR-L	720	**0.0041**	**3.4E-12**	910	**0.026**	0.056
PTR-R	644	**0.032**	**1.5E-12**	425	0.057	0.062
GCC	19	0.069	**1.8E-14**	1039	**0.031**	**0.022**
BCC	398	0.21	**1.6E-13**	1803	**0.046**	**0.012**
SCC	1010	**0.027**	**4.8E-12**	1654	**0.0012**	0.055
CGC-L	172	0.052	**2.4E-13**	73	**0.011**	**0.038**
CGC-R	2	0.41	**9.0E-14**	–	–	–
EC-L	602	**0.0058**	**2.5E-14**	60	**0.021**	**0.014**
EC-R	278	**0.033**	**5.3E-14**	370	**0.033**	**0.019**
SLF-L	98	**0.0019**	**5.9E-15**	549	**0.0059**	**0.012**
SLF-R	102	0.44	**4.9E-13**	161	**0.0077**	**0.010**
SS-L	156	**0.043**	**1.6E-13**	186	0.099	**0.013**
SS-R	187	0.17	**5.3E-13**	169	**0.021**	**0.043**

*The significance of group and FA to the GLMs are displayed. The significance of age and gender are described in the main text. ACR, anterior corona radiata; SCR, superior corona radiata; PCR, posterior corona radiata; ALIC, anterior limb of the internal capsule; PLIC, posterior limb of the internal capsule; RLIC, retrolenticular limb of the internal capsule; PTR, posterior thalamic radiation; GCC, genu of the corpus callosum; BCC, body of the corpus callosum; SCC, splenium of the corpus callosum; CGC, cingulate gyrus part of the cingulum; EC, external capsule; SLF, superior longitudinal fasciculus; SS, sagittal stratum. Bolded p values indicate statistically significant effects (p < 0.05).*

The significance of the group and FA predictor variables in the GLMs for prediction of the Sensory Profile tactile score and Graphesthesia are included in **Table 3**. The effects of group (TDC vs. SPD) for the Sensory Profile tactile score model were much more strongly significant than the group effects in the Graphesthesia model. The FA effect was significant for many ROIs for both the Sensory Profile tactile score and for Graphesthesia, though the effect was significant for more ROIs with Graphesthesia than with the Sensory Profile tactile score (12 ROIs for Sensory Profile tactile score, 16 ROIs for Graphesthesia). For the Sensory Profile tactile response variable, neither age nor gender was significant for any of the models. For graphesthesia, gender was significant for every model, with females achieving higher scores than males.

**FIGURE 3:**
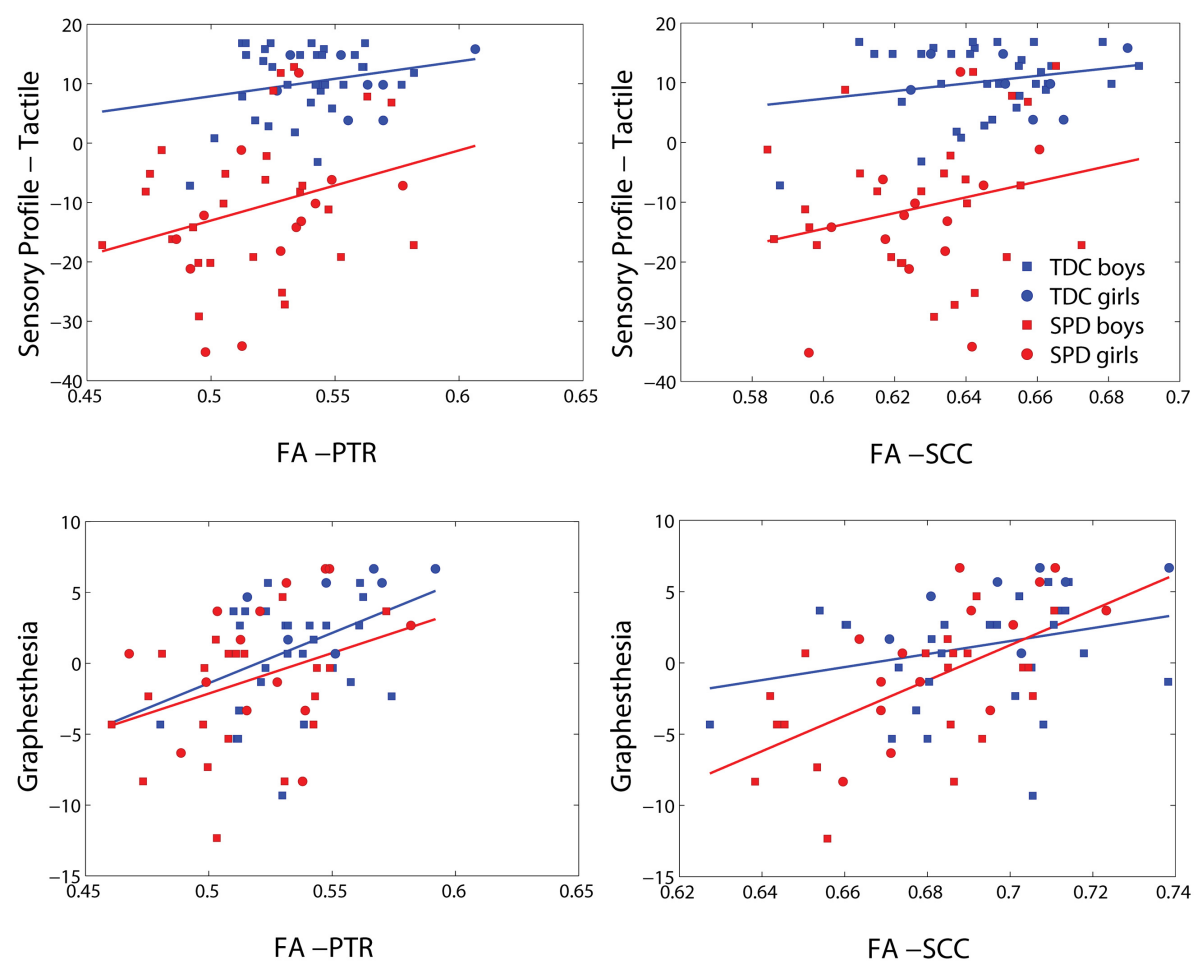
**Representative scatterplots and regression lines of the Sensory Profile tactile score and Graphesthesia (mean-centered) versus FA in significant voxels (differing between the two sensory variables) of the posterior thalamic radiations (PTRs) (both sides combined) and splenium of the corpus callosum (SCC)**.

Representative plots of the Sensory Profile tactile score model and Graphesthesia vs. FA in significant voxels of the bilateral PTR and SCC are displayed in **Figure 3**. The regression lines for TDC and SPD (of sensory score vs. FA) are more concordant with one another for Graphesthesia than for the Sensory Profile tactile score.

#### Auditory Correlations with DTI

**FIGURE 4:**
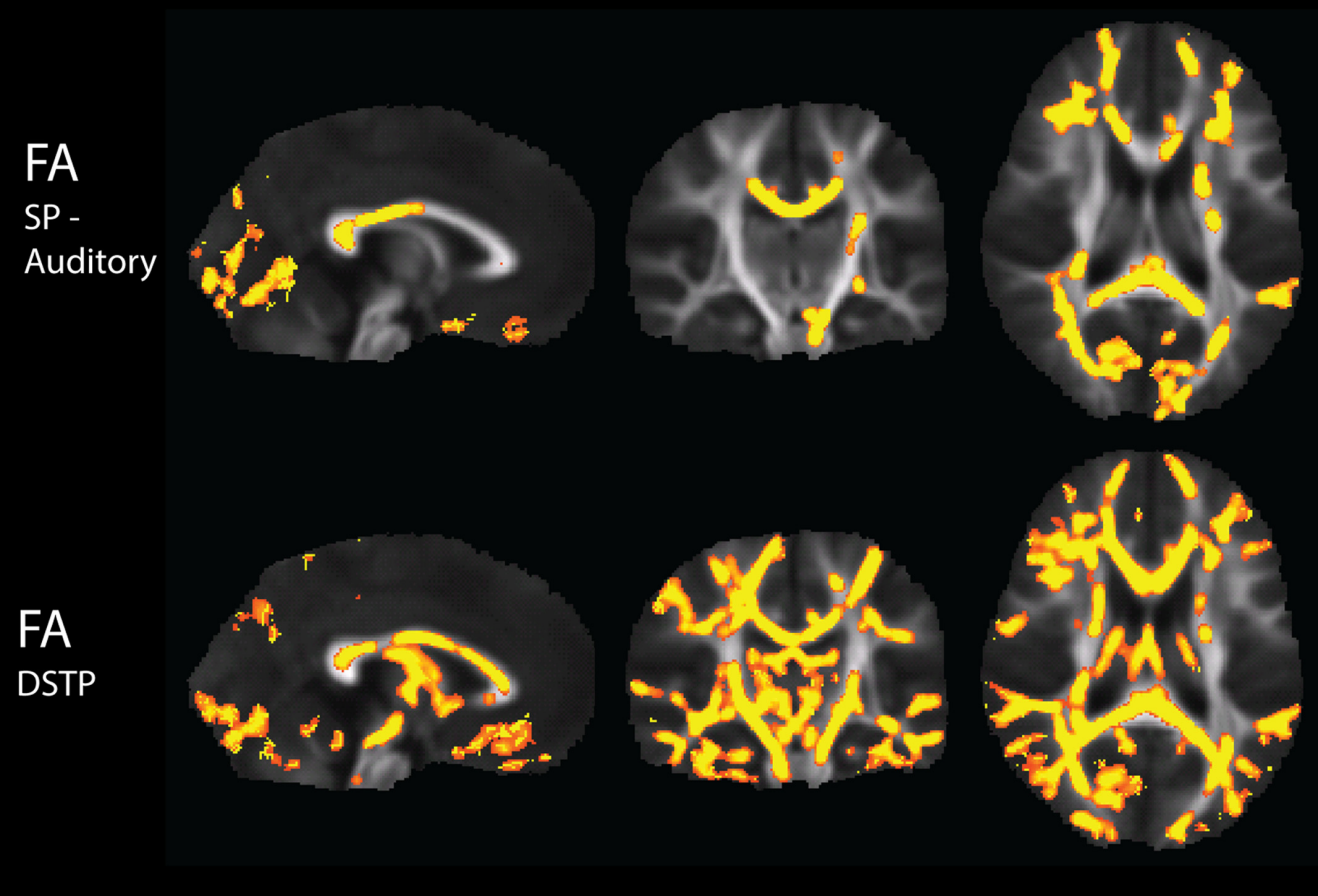
**Tract-Based Spatial Statistics results of correlations of the Sensory Profile auditory score and DSTP with FA, including regression of motion**.

There were widespread, significant positive associations of FA across groups with the Sensory Profile auditory score and with DSTP, after regression of motion parameters (**Figure 4**). The number of significantly correlated voxels in several ROIs is included in **Table 4**.

**Table 4:** Number of significantly correlated voxels in several ROIs, along with results of the GLMs of Sensory Profile auditory score and DSTP as functions of group, FA, age, and gender.

	SP – Auditory			DSTP		
	Num vox	p_FA	p_TDCvSPD	Num vox	p_FA	p_TDCvSPD
ACR-L	562	0.13	**9.9E-15**	1155	**0.014**	0.077
ACR-R	573	0.26	**9.7E-15**	1371	**0.00036**	0.12
SCR-L	79	0.17	**1.7E-15**	148	**0.067**	**0.038**
SCR-R	61	0.77	**2.5E-15**	464	**0.029**	**0.043**
PCR-L	48	0.26	**2.5E-15**	236	**0.081**	**0.050**
PCR-R	298	0.11	**4.8E-15**	518	**0.016**	0.068
ALIC-L	276	0.06	**1.1E-14**	398	**0.0074**	0.18
ALIC-R	374	0.20	**1.8E-14**	576	**0.010**	0.19
PLIC-L	339	0.28	**7.8E-16**	491	**0.016**	0.058
PLIC-R	173	0.64	**2.8E-15**	553	**0.011**	0.082
RLIC-L	–	–	–	539	**0.016**	0.075
RLIC-R	5	0.69	**6.4E-16**	475	**0.039**	0.11
PTR-L	396	**0.026**	**6.8E-14**	647	**0.029**	0.23
PTR-R	429	**0.029**	**6.5E-14**	768	**0.013**	0.29
GCC	551	0.25	**8.1E-15**	1234	**0.020**	0.086
BCC	1718	0.055	**9.2E-16**	2173	**0.048**	**0.046**
SCC	1293	0.096	**5.3E-14**	1032	**0.027**	0.18
CGC-L	102	**0.038**	**1.7E-15**	255	**0.022**	0.067
CGC-R	7	**0.021**	**3.9E-15**	10	0.26	**0.041**
EC-L	399	**0.00038**	**2.7E-17**	383	0.091	**0.048**
EC-R	289	**0.010**	**1.7E-16**	1104	**0.002**	0.063
SLF-L	–	–	–	560	**0.018**	0.068
SLF-R	141	0.062	**5.1E-15**	852	**0.00040**	0.060
SS-L	–	–	–	246	**0.0055**	**0.047**
SS-R	–	–	–	308	**0.00016**	0.36

*The significance of group and FA to the GLMs are displayed. The significance of age and gender are described in the main text. ACR, anterior corona radiata; SCR, superior corona radiata; PCR, posterior corona radiata; ALIC, anterior limb of the internal capsule; PLIC, posterior limb of the internal capsule; RLIC, retrolenticular limb of the internal capsule; PTR, posterior thalamic radiation; GCC, genu of the corpus callosum; BCC, body of the corpus callosum; SCC, splenium of the corpus callosum; CGC, cingulate gyrus part of the cingulum; EC, external capsule; SLF, superior longitudinal fasciculus; SS, sagittal stratum. Bolded p values indicate statistically significant effects (p < 0.05).*

The significance of the group and FA predictor variables in the GLMs for prediction of the Sensory Profile auditory score and DSTP are included in **Table 4**. Similarly to the tactile model, the group effects (TDC vs. SPD) in the Sensory Profile auditory score model were much more strongly significant than group effects in the DSTP model, with the DSTP group effects only reaching weak significance in a few ROIs. The FA effect was significant for many ROIs for DSTP, but only in a few ROIs for the Sensory Profile auditory score. For the Sensory Profile auditory response variable, neither age nor gender was significant for any of the models. For DSTP, age was significant for every model, with increasing age giving rise to higher scores.

**FIGURE 5:**
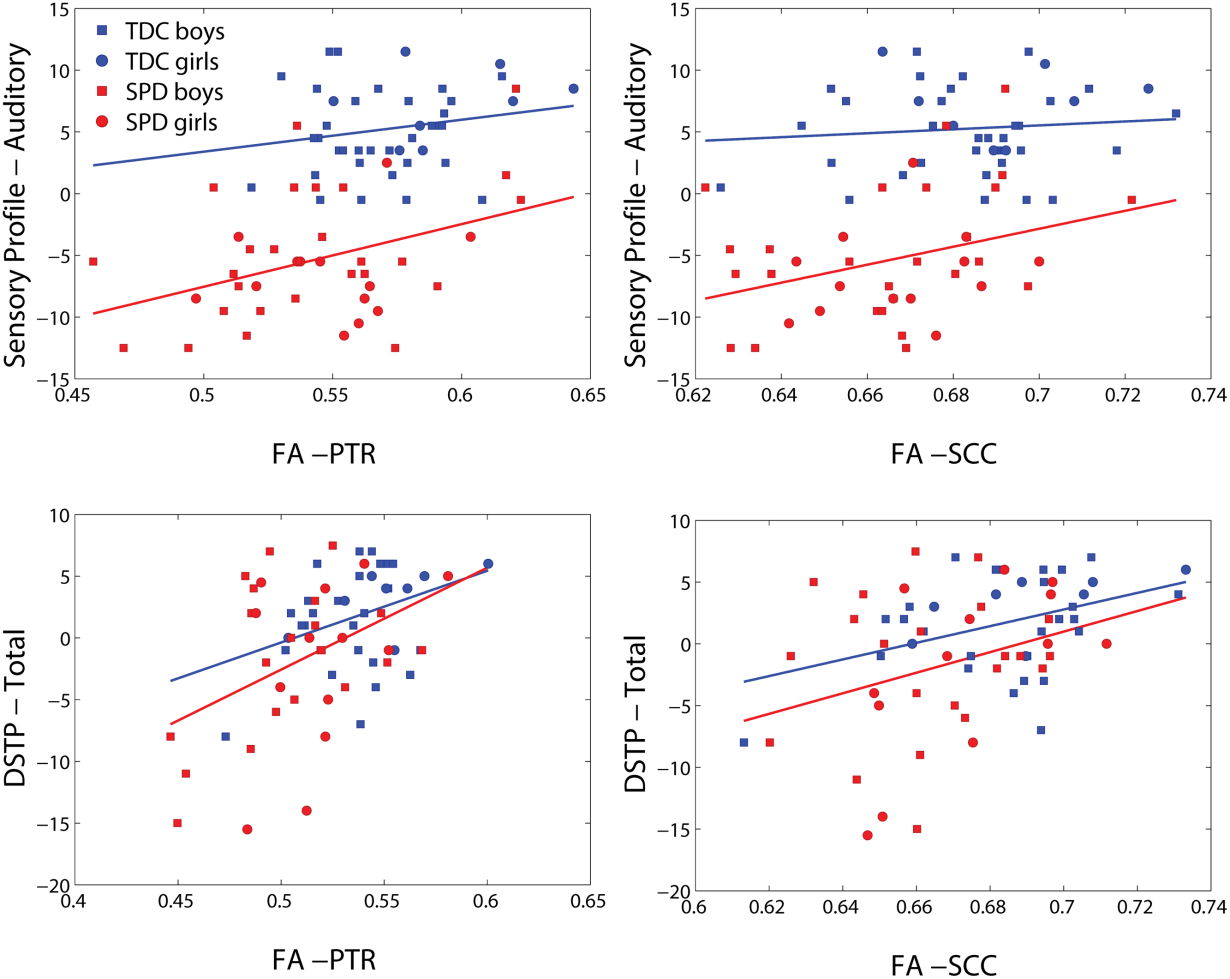
**Representative scatterplots and regression lines of the Sensory Profile auditory score and DSTP (mean-centered) versus FA in significant voxels (differing between the two sensory variables) of the posterior thalamic radiations (PTR) (both sides combined) and splenium of the corpus callosum (SCC)**.

Representative plots of the Sensory Profile tactile score model and Graphesthesia versus FA in significant voxels of the bilateral PTR and SCC are displayed in **Figure 5**. The regression lines for TDC and SPD (of sensory score vs. FA) are more concordant with one another for Graphesthesia than for the Sensory Profile tactile score.

#### DSTP Subscore Correlations with DTI

Given the extensive and robust correlations of FA with the DSTP acoustic subscore, additional post-hoc tests for correlations were performed between FA and the three subscores of the DSTP acoustic test – dichotic digits, temporal patterning, and auditory discrimination. The number of significantly correlated voxels in several ROIs is included in **Table 5**. Overall, the dichotic digits task was significantly associated with the largest number of voxels, as compared with temporal patterning and auditory discrimination.

**Table 5:** Number of significantly correlated voxels in several ROIs, along with results of the three DSTP subscores as functions of group, FA, age, and gender.

	DSTPdd			DSTPtp			DSTPad		
	# sig vox	p_FA	p_TDCvSPD	# sig vox	p_FA	p_TDCvSPD	# sig vox	p_FA	p_TDCvSPD
ACR-L	734	**0.016**	0.32	582	0.10	**0.010**	–	–	–
ACR-R	1190	**0.0025**	0.34	918	**0.028**	**0.011**	1176	**0.00057**	0.52
SCR-L	334	**0.037**	0.15	7	0.23	**0.0022**	–	–	–
SCR-R	460	**0.022**	0.18	326	**0.037**	**0.0033**	83	**0.029**	0.19
PCR-L	255	**0.013**	0.21	52	0.27	**0.0061**	–	–	–
PCR-R	499	**0.0034**	0.27	341	**0.046**	**0.0086**	40	**0.012**	0.25
ALIC-L	268	**0.038**	0.36	316	**0.0074**	**0.021**	–	–	–
ALIC-R	521	**0.013**	0.50	475	**0.037**	**0.028**	326	**0.0054**	0.64
PLIC-L	584	**0.0050**	0.21	306	0.077	**0.0048**	–	–	–
PLIC-R	602	**0.0025**	0.30	338	0.29	**0.0070**	18	**0.0063**	0.55
RLIC-L	455	**0.0091**	0.28	440	**0.043**	**0.0087**	–	–	–
RLIC-R	453	**0.0083**	0.43	363	0.14	**0.012**	111	**0.026**	0.62
PTR-L	596	**0.0075**	0.67	463	0.32	**0.019**	–	–	–
PTR-R	894	**0.00043**	0.99	583	0.12	**0.031**	305	**0.0072**	0.81
GCC	1116	**0.014**	0.33	928	0.095	**0.0088**	426	**0.043**	0.44
BCC	2048	**0.030**	0.18	1788	0.085	**0.0044**	529	**0.041**	0.23
SCC	939	**0.028**	0.43	871	0.074	**0.021**	129	**0.025**	0.45
CGC-L	268	**0.0064**	0.28	153	**0.025**	**0.0091**	–	–	–
CGC-R	8	**0.11**	0.20	69	0.080	**0.0080**	–	–	–
EC-L	223	**0.13**	0.18	439	**0.049**	**0.0041**	–	–	–
EC-R	911	**0.0054**	0.23	598	**0.013**	**0.0045**	752	**0.00054**	0.44
SLF-L	702	**0.0014**	0.24	9	0.14	**0.0024**	–	–	–
SLF-R	981	**0.00046**	0.18	715	**0.014**	**0.0067**	206	**0.0013**	0.45
SS-L	177	**0.023**	0.20	235	**0.0068**	**0.0051**	5	**0.0026**	0.18
SS-R	259	**0.0054**	0.56	284	**0.016**	**0.032**	212	**0.00044**	0.98

*The significance of group and FA to the GLMs are displayed. The significance of age and gender are described in the main text. DSTPdd, dichotic digits; DSTPtp, temporal patterning; DSTPad, auditory discrimination. ACR, anterior corona radiata; SCR, superior corona radiata; PCR, posterior corona radiata; ALIC, anterior limb of the internal capsule; PLIC, posterior limb of the internal capsule; RLIC, retrolenticular limb of the internal capsule; PTR, posterior thalamic radiation; GCC, genu of the corpus callosum; BCC, body of the corpus callosum; SCC, splenium of the corpus callosum; CGC, cingulate gyrus part of the cingulum; EC, external capsule; SLF, superior longitudinal fasciculus; SS, sagittal stratum. Bolded p values indicate statistically significant effects (p < 0.05).*

The significance of the group and FA predictor variables in the GLMs for prediction of each DSTP subscore are included in **Table 5**. The group effect (TDC vs. SPD) was only significant for the temporal patterning task, while the FA effect was significant for all tasks, but most strongly for dichotic digits. For the dichotic digits test and temporal patterning test, age was a significant contributor for most models, with increasing age giving rise to higher scores, but gender did not contribute significantly to the model. For auditory discrimination, neither age nor gender was significant.

**FIGURE 6:**
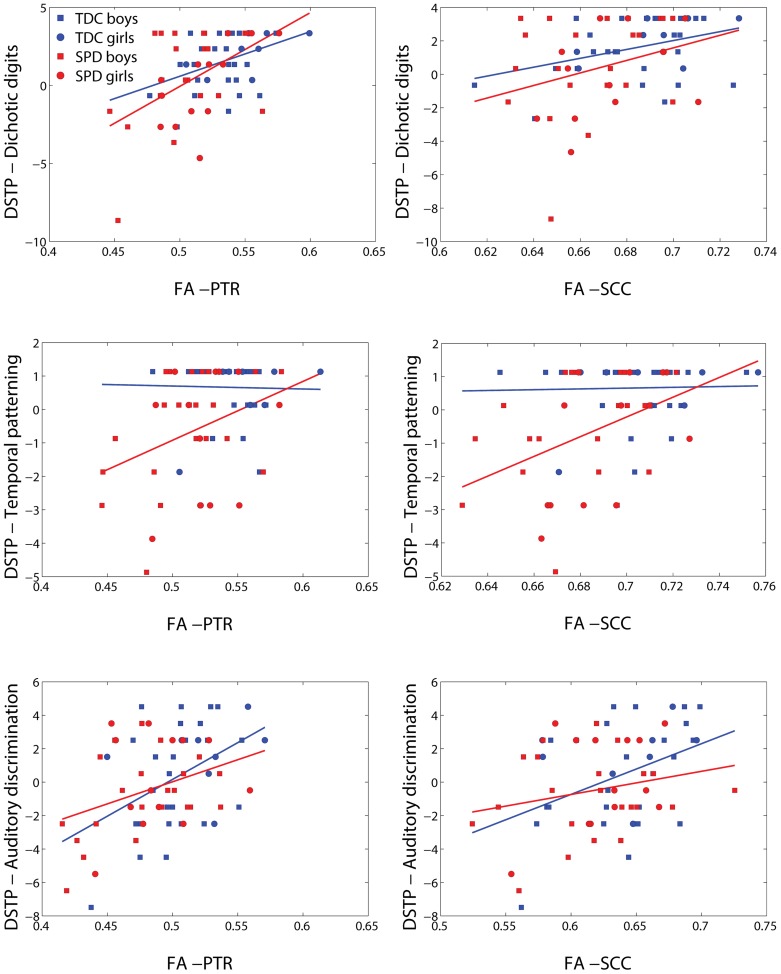
**Representative scatterplots and regression lines of the DSTP subscores (mean-centered) – dichotic digits, temporal patterning, and auditory discrimination – vs. FA in significant voxels (differing between the three sensory variables) of the PTR (both sides combined) and splenium of the corpus callosum (SCC)**.

Representative plots of each DSTP subscore versus FA in significant voxels of the bilateral PTR and SCC are displayed in **Figure 6**. The regression lines for TDC and SPD (of sensory score vs. FA) are concordant with one another for dichotic digits and auditory discrimination, but not temporal patterning. A ceiling effect of the dichotic digits and temporal patterning tasks can be observed from these plots, limiting the range of sensory function that can be differentiated between subjects, particularly in the TDC cohort.

## Discussion

These findings corroborate and generalize our previous work demonstrating the role of disrupted posterior white matter microstructure in SPD. Furthermore, the larger, mixed-gender cohort unmasks a more extensive distribution of white matter differences which includes anterior white matter. More importantly, to our knowledge, this work is the first to demonstrate a relationship between direct measurements of tactile function and non-linguistic auditory function with white matter microstructural integrity not just in SPD, but also in TDC.

### Group Differences

As in our prior work (Owen et al., 2013), our results show strong decreases of white matter microstructural integrity in the posterior projection and commissural tracts of the bilateral PTR and the SCC, which respectively contain all primary sensory projection pathways excluding olfaction, and connect homologous sensory cortical regions (Huang et al., 2005; Hofer and Frahm, 2006). Our findings additionally reveal marked reductions of microstructural integrity in the RLIC, which contains visual and auditory projection fibers. Overall, microstructural integrity is disrupted extensively, but with a posterior bias, throughout the white matter.

### Sensory Correlations with DTI

We find that correlations of white matter microstructure with direct measurements of tactile and auditory processing are stronger than the correlations of microstructure with parent report measures, likely due to the more objective nature of the direct measurements. Furthermore, the stronger concordance between the TDC and SPD regression lines of FA with the direct sensory measurements of Graphesthesia and DSTP, as compared with the sensory profile tactile and auditory scores, suggest that these direct measurements map more closely to the underlying biology. The offset between the TDC and SPD regression lines of the sensory profile metrics with FA may reflect biased parent reporting as a function of the presence or absence of an SPD diagnosis. More explicitly, if a child without an SPD label and a child with an SPD label exhibit the same level of function for a given sensory processing domain, parents of the child who has not been attributed an SPD label may be less likely to report abnormalities than the parents of the child who has been clinically assigned an SPD label.

The relative lack of anatomic specificity of these correlations of DTI with sensory processing measures may be due, in part, to the high degree of microstructural covariance between different white matter tracts (Wahl et al., 2010), with a spatially global factor accounting for almost half of the variance in FA (Penke et al., 2010). It is also likely reflective of the multiple functional brain networks required for each of these tests, as reviewed below.

#### Tactile Processing

Diffusion tensor imaging has previously been used to link degree of periventricular white matter injury in the PTR, as assessed by size reduction of white matter tracts on visual inspection, with contralateral touch threshold, proprioception, and motor severity in children with cerebral palsy (Hoon et al., 2009). However, this prior work used a subjective rater system of white matter injury. One study has reported a correlation between tactile defensiveness and FA in the inferior longitudinal fasciculus of children with ASD (Pryweller et al., 2014). However, the metric of tactile defensiveness is an assessment of sensory-related behavior, rather than early-stage sensory function. While we are not aware of other studies reporting a relationship between white matter microstructure and primary or secondary tactile processing, there are a multitude of functional imaging studies have demonstrated primarily fronto-parietal network activations in tasks involving either active or passive touch discrimination of macrogeometric object features such as shape or length (Bodegård et al., 2001; Van de Winckel et al., 2005). These circuits include the ventral and dorsal premotor cortex, secondary somatosensory area, superior parietal lobe, anterior part of the intraparietal sulcus (AIP), and supramarginal gyrus (Kawashima et al., 1994; O’Sullivan et al., 1994; Hadjikhani and Roland, 1998; Roland and Zilles, 1998; Binkofski et al., 1999; Bodegård et al., 2000, 2001; Servos et al., 2001; Stoeckel et al., 2003, 2004; Stoesz et al., 2003).

Our results are the first to demonstrate associations of white matter microstructure with tactile processing, both among children with SPD as well as among TDC. Both the Sensory Profile tactile score and Graphesthesia are associated with FA in regions subserving primary sensory processing, including the posterior projection tracts of the superior and posterior corona radiata, the posterior limbs of the internal capsule, and the PTR. Both are also associated with the SCC, which connects homologous sensory areas. However, they are also associated with microstructure in associational tracts such as the external capsules, superior longitudinal fasciculus, sagittal stratum. Unlike the Sensory Profile tactile score, Graphesthesia demonstrates associations with the frontal regions of the right anterior corona radiata, anterior limb of the RLIC, and the genu of the corpus callosum. The widespread nature of these correlations may in part reflect the non-specificity of the assessments used, in addition to the previously mentioned contribution of high microstructural covariance of white matter (from the beginning of the Discussion). For example, in addition to primary tactile processing, Graphesthesia engages secondary modalities requiring synthesis and interpretation of the primary tactile inputs, including the spatio-temporal analysis of these inputs to form a visual representation of what was drawn on the back of the hand, and the motor planning and coordination to re-create this image.

The tactile sense develops the earliest among all sensory systems, with somatosensory responses detected *in utero* as early as 8 weeks of gestation (Montagu, 1986). Tactile feedback from mechanoreceptors in the skin and joints critically guide the development of motor skills during childhood, both in gross motor (Metcalfe et al., 2005) and fine motor (Soechting and Flanders, 2008) functions. Tactile processing has been further suggested to play a central role in the development of social and communicative behaviors, with tactile-centered therapies shown to effectively modulate arousal, attention, and sensory defensiveness (Cascio, 2010). It is thus critical to understand the neural correlates of tactile processing in order to better understand the downstream effects of its abnormalities as well as to better design and evaluate tactile-centered therapies.

#### Auditory Processing

One prior study reported associations between FA and performance on auditory processing tasks in TDC (Schmithorst et al., 2011). This prior study reports positive correlations of FA around the prefrontal regions with performance in a speech-in-noise task. However, all measures of auditory processing in this previous study involved manipulations of language stimuli, unlike the acoustic test of the DSTP which involves manipulations of non-linguistic stimuli, therefore being more likely to probe basic auditory processing function instead of the integrity of language networks.

To our knowledge, our study is the first to demonstrate associations of white matter microstructure with non-linguistic auditory processing, both among children with SPD as well as among TDC. Both the Sensory Profile auditory score and DSTP are associated with FA in the PTR, which contains the primary auditory projection pathway. However, they are also associated with microstructure in associational tracts such as the external capsules and the cingulate portion of the cingulum bundles. Unlike the Sensory Profile auditory score, DSTP demonstrates widespread associations with both frontal and posterior projection and commissural pathways, along with the associational tracts of the superior longitudinal fasciculus and sagittal stratum. Similar to tactile processing, one contributor to the extensive regions of significant correlations with the DSTP task may lie in the test’s additional recruitment of attentional processes. For example, dichotic listening tasks have long been used to test different neural models of attention (Broadbent, 1954; Treisman, 1964), and have been used to demonstrate enhanced right ear advantage in individuals with mild Alzheimer’s disease (Duchek and Balota, 2005), decreased right ear advantage in children with dyslexia (Helland and Asbjornsen, 2001), and challenges with attention shifting in sleep deprivation (Johnson et al., 2002). The dichotic listening portion of the DSTP demonstrates the most robust correlations with FA of the three DSTP subtests, showing strong FA effects across projection, commisural, and association tracts (**Table 5**). Temporal patterning shows strong effects in certain projection and association tracts. It has previously been suggested that children with specific language impairment perform worse on temporal patterning tasks, but only when the interstimulus interval between tones is short (Tallal and Piercy, 1973). Interestingly, the left RLIC, with which temporal patterning demonstrates an FA effect in our present study (but not with the right RLIC), has been previously associated with reading scores in individuals with dyslexia by a DTI study (Klingberg et al., 2000). Auditory discrimination, which involves a strong element of attentional control, demonstrates the strongest FA effects with the right anterior corona radiata, which is concordant with Schmithorst et al.’s (2011) finding of correlations of language discrimination in noise with FA in white matter of prefrontal regions.

Auditory processing is of primary importance for language acquisition, with speech perception requiring the ability to determine spectral shape, to discriminate modulation of amplitude and spectral frequencies, and to do this at varying temporal resolutions (Bailey and Snowling, 2002). These auditory capabilities are likely sufficient by early infancy to support the discrimination of phonetic elements in language, though attentional capabilities may take longer to develop (Bailey and Snowling, 2002). Given the wealth of evidence that developmental language-based learning disorders can often be traced to non-verbal auditory processing deficits (Tallal et al., 1997), it is highly relevant to characterize the biological underpinnings of these deficits.

### Study Limitations and Future Directions

Despite the larger number of subjects in this study, we are still limited by sample size in our ability to harness DTI as a clinically utilizable tool for the diagnosis, prognosis, and treatment of SPD. Going forward, larger sample sizes and multimodal imaging biomarkers from DTI, fMRI, and MEG may aid in better definition and diagnosis of SPD. This could be of particular use if these biomarkers can identify individuals at risk for SPD at early ages before clinical symptoms are apparent, allowing for early intervention and recruitment of support services. In addition to diagnosis, larger scale longitudinal studies will allow us to evaluate the utility of quantitative imaging biomarkers, as compared with clinical metrics, neuropsychological testing, or direct sensory testing, for the prognostication of behavioral and cognitive development of individuals with SPD. Finally, interventional studies will allow us to evaluate the utility of quantitative imaging biomarkers for the monitoring of behavioral and psychopharmacological interventions, as well as for the prediction of interventional outcome. These biomarkers may further aid in the design of interventions if they can be used to stratify the SPD population into subgroups that will better respond to particular interventions. Overall, future studies will aim to shift SPD from a clinical diagnosis to a “biomarker diagnosis,” with imaging, and in particular DTI, metrics among the most promising of these biomarkers.

## Author Contributions

Conception and design: JO, EM, PM. Acquisition: AB-A, SD, SH, ABA, JH. Analysis: Y-SC, MG, JO, EM, PM. Interpretation: YSC, MG, JPO, EJM, PM.

## Conflict of Interest Statement

The authors declare that the research was conducted in the absence of any commercial or financial relationships that could be construed as a potential conflict of interest.

## Footnotes

1 www.fmrib.ox.ac.uk/fsl/flirt

2 http://www.fmrib.ox.ac.uk/analysis/research/bet
